# Surgery for gastric cancer in a patient with non-cirrhotic hyperammonemia: a case report

**DOI:** 10.1186/s12957-015-0500-2

**Published:** 2015-02-22

**Authors:** Bo Liu, Miao Yu, Yong-xi Song, Peng Gao, Hui-mian Xu, Zhen-ning Wang

**Affiliations:** Department of Surgical Oncology and General Surgery, First Hospital of China Medical University, 155 North Nanjing Street, Heping District, Shenyang City, 110001 China

**Keywords:** Spontaneous portacaval shunt, Gastric cancer, Hyperammonemia, Gastrectomy

## Abstract

We report a case of gastric cancer in a patient with non-cirrhotic hyperammonemia secondary to a spontaneous portacaval shunt. The patient, a 69-year-old male, had more than 40 years of abdominal discomfort. On gastroscopy, 2.0 × 1.5-cm irregular uplift ulcers were seen on the lesser curvature of the stomach, and tissue biopsy revealed poorly differentiated adenocarcinoma. His hyperammonemia was found on celiac angiography to be due to the formation of a spontaneous portacaval shunt. Imaging revealed no evidence of cirrhosis or portal hypertension. The patient ultimately underwent a distal gastrectomy and gastroduodenal anastomosis; the spontaneous portacaval shunt was left untreated. Postoperatively, there were no short-term complications such as anastomotic leakage, stricture, or bleeding, and the patient’s blood ammonia level decreased to within the normal range. Radical gastrectomy without splenectomy or closure of the abnormal shunt was feasible for the treatment of gastric cancer in a patient with non-cirrhotic hyperammonemia.

## Background

Hyperammonemia is associated with high perioperative morbidity and mortality in patients undergoing gastric surgery. The operative indications are difficult to determine for gastric cancer patients with hyperammonemia. We report a case of surgical treatment for gastric cancer in a patient with hyperammonemia.

## Case presentation

A 69-year-old man with a history of hyperammonemia presented to the First Hospital of China Medical University with more than 40 years of abdominal discomfort. This discomfort was described as having no regular pattern or relationship to diet and was slightly relieved by oral omeprazole. He underwent gastroscopy at a local hospital that revealed 2.0 × 1.5-cm irregular uplift ulcers in the lesser curvature of the stomach. The tissue was friable, bled easily, and had a dirty-moss appearance and surrounding mucosal edema. And, peristalsis had not been present. Tissue biopsy revealed poorly differentiated adenocarcinoma.

His medical history included hypertension for 20 years and coronary heart disease for more than 10 years. Two years earlier, the patient had been diagnosed with a spontaneous portacaval shunt with recurrent headache and an extremely elevated blood ammonia concentration. He had no history of diabetes, chronic hepatitis, or tuberculosis and no surgical or trauma history.

The patient was in good overall condition. His vital signs were stable and his weight was stable. His skin and mucous membranes were not scleral icterus. He had no liver palms or spider angiomata. His abdomen was flat and symmetric, without peristaltic wave, or caput medusae. He had normal bowel sounds without succussion splash or vascular murmur and no abdominal tenderness or rebound tenderness. No mass was palpated in the liver or spleen. There was no shifting dullness.

Laboratory examination demonstrated no anemia and a carbohydrate antigen 19-9 (CA 19-9) of 55.73 U/mL (reference range 0 to 27 U/mL). The patient’s blood ammonia concentration was 123 μmol/L, which was significantly higher than the upper limit of normal (reference range 9 to 33 μmol/L). Other laboratory values were within the normal range.

**Figure 1:**
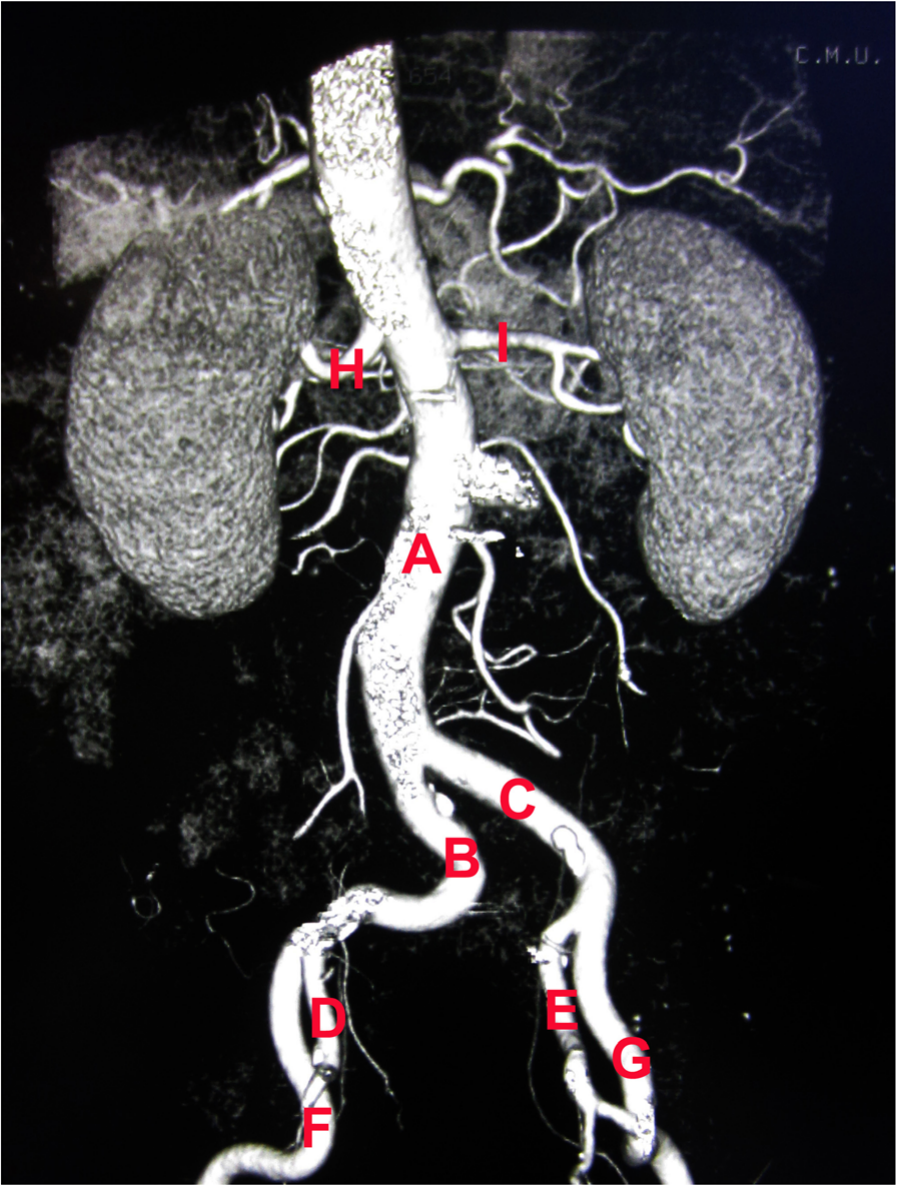
**Abdominal aortic CT.** (A) Abdominal aorta. (B) Right common iliac artery. (C) Left common iliac artery. (D) Right internal iliac artery. (E) Left internal iliac artery. (F) Right external iliac artery. (G) Left external iliac artery. (H) Right renal artery. (I) Left renal artery.

Enhanced three-dimensional computed tomography (3D-CT) showed gastric wall thickening, an uneven mucosal surface, and ulcer formation at the gastric angle. There were clear boundaries between thickened and normal areas of the gastric wall. The gastric serosal surface was smooth. Unenhanced CT scan demonstrated a thickened parietal layer with a value of about 36 Hounsfield units, which increased after enhancement. There was no lymph node enlargement in the peri-gastric or peri-aortic areas, including the posterior abdominal wall and inferior vena cava, and no abnormal soft tissue density in the peritoneum, omentum, or mesentery. On unenhanced CT, multiple round hypodensities were seen in the liver. Color Doppler ultrasound did not demonstrate any abnormalities in the gallbladder, spleen, or pancreas. Abdominal aortic CT showed that the walls of the abdominal aorta, bilateral common iliac arteries, and bilateral internal iliac arteries were not smooth, and multiple punctate calcifications were seen in the local vascular walls. There was no significant luminal narrowing. The celiac trunk and branches of the superior mesenteric artery, bilateral renal arteries, and superior mesenteric arteries were normal (Figure 1).

**Figure 2:**
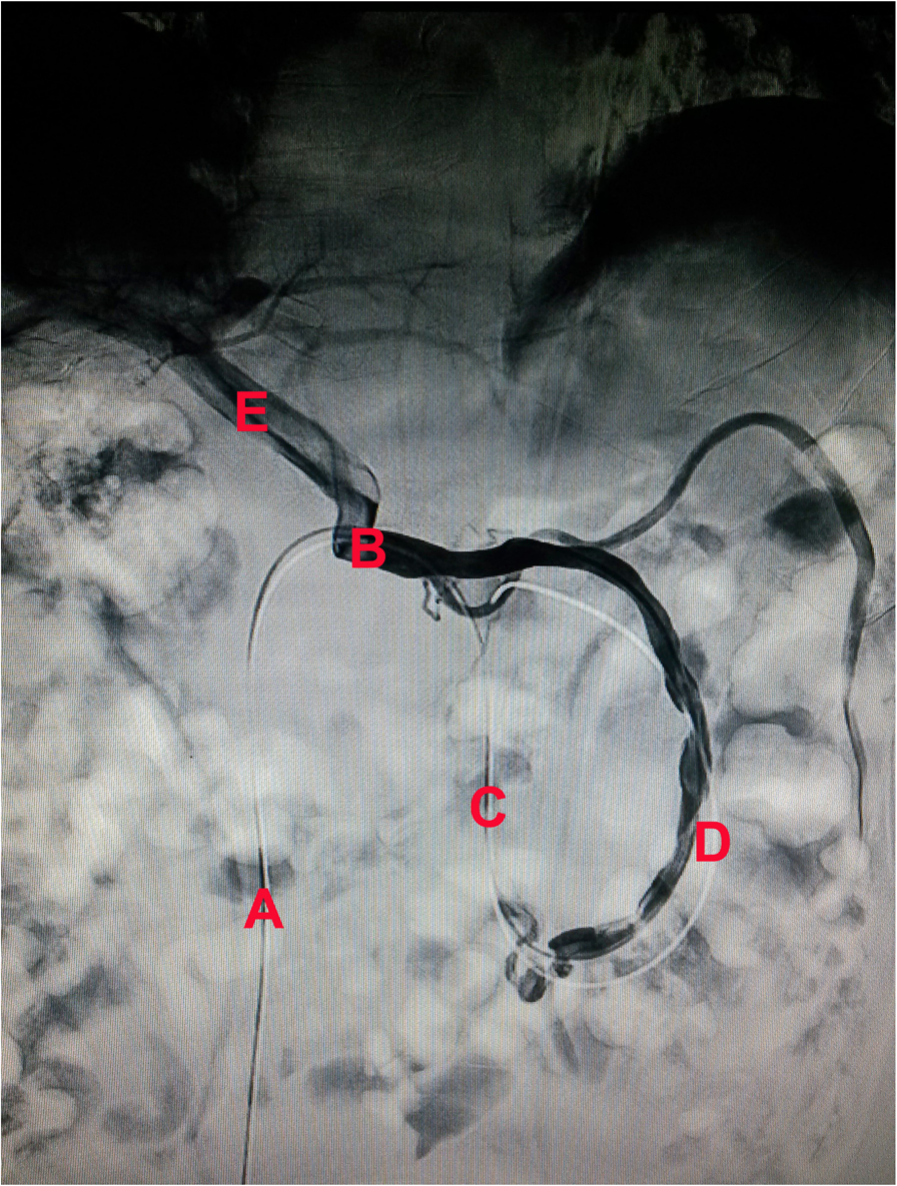
**Celiac angiography.** (A) Inferior vena cava. (B) Left renal vein. (C) Spermatic vein. (D) Superior mesenteric vein. (E) Portal vein.

After discussion in the surgical oncology department, the patient underwent celiac angiography. During the procedure, we observed an abnormal communication of the superior mesenteric vein with the spermatic vein, which demonstrated compensatory enlargement and an abnormal shunt direction from the portal vein to the superior mesenteric vein, spermatic vein, left renal vein, and inferior vena cava. After successful puncture of the right femoral vein using Seldinger technique, a super-smooth guidewire with a Cobra tip (Terumo Interventional Systems, Somerset, NJ, USA) was advanced to the left renal vein, then into the portal vein via the abnormal shunt. Portal vein and superior mesenteric vein angiography was performed using a high-pressure syringe, with smooth delivery of contrast agent. No clear stenosis was seen (Figure 2). Therefore, this abnormal shunt was considered congenital condition and no treatment. The patient was given lactulose 10 g orally twice a day and ornithine aspartate 15 g intravenous infusion once a day, for 7 days prior to surgery to reduce blood ammonia. He also received two water enemas the night before the operation.

**Figure 3:**
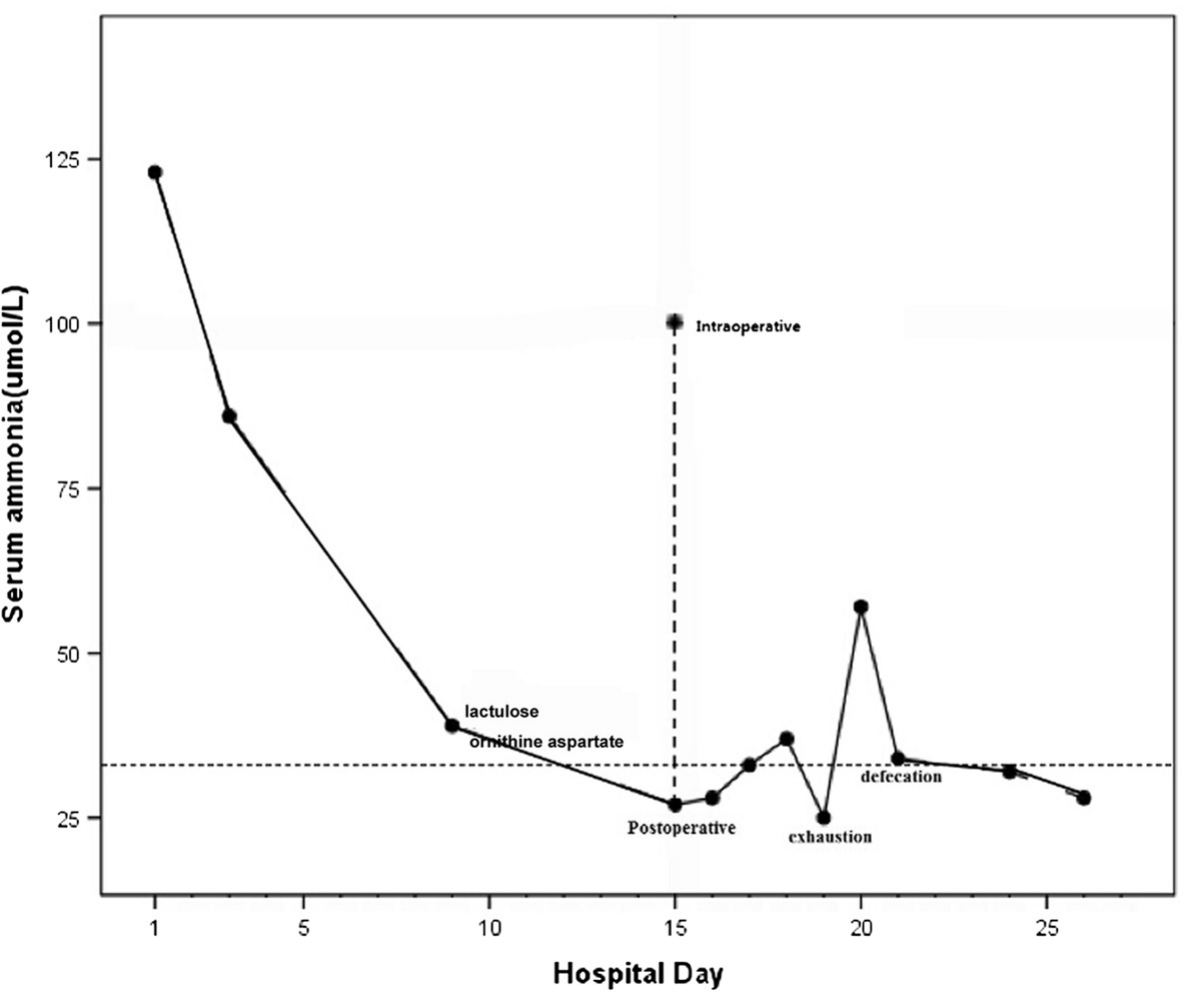
**Blood ammonia value changed during hospitalization.** The blood ammonia value was 123 μmol/L when the patient admitted to the hospital. And, it was delined from 100 to 27 μmol/L during the operation. And, it had a one-time increase postoperative, and then it was normal.

At a multidisciplinary meeting, we discussed performing interventional embolization of the shunt if ammonia continued to be elevated after gastrectomy in this elderly patient with normal liver function and no cirrhosis. The patient underwent radical subtotal resection of the distal stomach under general anesthesia without splenectomy or closure of the abnormal shunt. Intraoperative exploration revealed a lesion in the lesser curvature of the gastric body. Postoperative pathology revealed a 3.5 × 3 × 1.5-cm main focus located primarily in the lesser curvature of the gastric body. General classification: Borrmann III; histological type: poorly differentiated adenocarcinoma and signet ring-cell carcinoma; infiltration depth: subepithelial; growth pattern: diffuse growth; vein thrombosis: none; lymphatic invasion: none; residual stump: none; lymph node metastases: none (0/18). The patient recovered well postoperatively. Blood ammonia was well controlled and within the normal range (Figure 3). The patient was discharged on postoperative day 12. At 3-month follow-up, no abnormalities were seen on physical or laboratory examination. In particular, blood ammonia concentration continued to be within the normal range.

## Discussion

Gastric cancer is one of the most common gastrointestinal cancers worldwide, with a high incidence and cancer-related mortality [1]; radical gastrectomy remains the primary cure [2]. Not only hyperammonemia is commonly found in patients with liver failure because of the reduced ability of the liver to synthesize urea, but it is also seen in patients with portosystemic branch circulation, in which increases in intestinal ammonia have direct access to the systemic circulation, thus elevating blood ammonia. Encephalopathy, including recurrent headache, acute mental status changes, asterixis, tremor, and consciousness, is the main complication of the hyperammonemia in patients with and without liver cirrhosis [3]. We have reported the case of a 69-year-old man with gastric cancer and non-cirrhotic hyperammonemia.

To prevent encephalopathy and reduce perioperative risks during radical gastrectomy, it is important to determine the etiology of the hyperammonemia preoperatively. Ultrasonography was shown to be a reliable and noninvasive diagnostic method in 90 dogs with hyperammonemia [4]. Ubara *et al*. reported the use of color Doppler ultrasonography, in a hyperammonemic patient without liver function abnormalities, to identify a large shunt between the left gastric vein and left renal vein leading to portal flow entering the systemic circulation via the renal vein [5]. However, color Doppler ultrasonography of our patient demonstrated a portal system without expansion and revealed no abnormal shunt. Ultimately, percutaneous angiography revealed an unusual diversion that was the most likely cause of elevated blood ammonia.

Following radical gastrectomy, the patient’s blood ammonia decreased significantly to within the normal range, where it remained at 3-month follow-up. We believe this supports the effect of gastric cancer on blood ammonia levels in our patient without cirrhosis; however, this will require further study.

After clarifying the source of hyperammonemia, appropriate treatment may not only help control blood ammonia to prevent hepatic encephalopathy but also could reduce perioperative risk and extend the lives of patients with gastric cancer. Takeda *et al*. reported spontaneous portacaval shunts in two patients with gastric cancer and hepatic cirrhosis; one underwent a proximal gastrectomy and end-to-side esophagogastrostomy with splenectomy, including the closure of the shunt, and the other underwent a distal gastrectomy with devascularization around the gastric cardia, including splenectomy, with preservation of the short gastric vessels [6]. In another report, a 61-year-old woman with gastric cancer and a large-caliber portohepatic venous shunt underwent left lateral hepatic segmentectomy and subtotal gastrectomy with splenectomy [7]. Fenves *et al*. reported on five women with no history of liver disease who developed fatal hyperammonemic encephalopathy after Roux-en-Y gastric bypass surgery [8]. Our patient also underwent a distal gastrectomy and gastroduodenal anastomosis (Billroth I procedure); however, we did not perform a splenectomy or specific treatment for the spontaneous portacaval shunt. While the patient had no history of cirrhosis or portal hypertension, this abnormal shunt could have been addressed with interventional embolization if the hyperammonemia had persisted after gastric resection. Although the patient had an abnormal shunt, the gastric structure was normal. After the gastrectomy, there were no short-term complications of anastomotic leakage, stricture, or bleeding, and blood ammonia remained within the normal range, except for transient increases before the defecation. We believe this may be a feasible surgical approach in these patients.

Postoperative treatment continues to be important for gastric cancer patients with hyperammonemia. The patient may develop distant metastases postoperatively if the patient has portal vein metastasis and thrombosis, which may aggravate hyperammonemia and induce encephalopathy [9]; however, postoperative chemotherapy should not include 5-fluorouracil for these patients. A number of studies have shown that patients with advanced gastric cancer who received 5-fluorouracil-based chemotherapy may develop with mild toxicity, including hyperammonemia, which may result in hyperammonemia-related death [10-13]. Subsequent treatment must therefore be undertaken with caution.

## Conclusions

The most important factor affecting the prognosis for a hyperammonemia in patients with gastric cancer without hepatic cirrhosis is a definitive preoperative diagnosis. It is also important to determine which type of operation should be performed and to use medications such as oral lactulose and ornithine aspartate to lower blood ammonia in high-risk patients. Finally, gastrectomy without splenectomy or closure of the shunt is feasible.

## Consent

The patient signed an informed consent for the publication of this report and any concomitant images.

## Abbreviations

CA19-9: carbohydrate antigen 19-9
3D-CT: Three-dimensional computed tomography
CT: Computed tomography
